# Persistent Cutaneous *Leishmania major* Infection Promotes Infection-Adapted Myelopoiesis

**DOI:** 10.3390/microorganisms10030535

**Published:** 2022-02-28

**Authors:** Fabio Luiz Bandeira Ferreira, Olivier Séguin, Albert Descoteaux, Krista M. Heinonen

**Affiliations:** 1 Centre Armand Frappier Santé Biotechnologie, Institut National de la Recherche Scientifique, Laval, QC H7V 1B7, Canada; fabio.bandeira@inrs.ca (F.L.B.F.); olivier.seguin@inrs.ca (O.S.); albert.descoteaux@inrs.ca (A.D.); 2 Centre d’Excellence en Recherche sur les Maladies Orphelines-Fondation Courtois (CERMO-FC), Montreal, QC H2X 3Y7, Canada

**Keywords:** hematopoietic stem/progenitor cell, *Leishmania major*, cutaneous leishmaniasis, myelopoiesis, inflammation

## Abstract

Hematopoietic stem/progenitor cells (HSPC) are responsible for the generation of most immune cells throughout the lifespan of the organism. Inflammation can activate bone marrow HSPCs, leading to enhanced myelopoiesis to replace cells, such as neutrophils, which are attracted to inflamed tissues. We have previously shown that HSPC activation promotes parasite persistence and expansion in experimental visceral leishmaniasis through the increased production of permissive monocytes. However, it is not clear if the presence of the parasite in the bone marrow was required for infection-adapted myelopoiesis. We therefore hypothesized that persistent forms of *Leishmania major* (cutaneous leishmaniasis) could also activate HSPCs and myeloid precursors in the C57Bl/6 mouse model of intradermal infection in the ear. The accrued influx of myeloid cells to the lesion site corresponded to an increase in myeloid-biased HSPCs in the bone marrow and spleen in mice infected with a persistent strain of *L. major*, together with an increase in monocytes and monocyte-derived myeloid cells in the spleen. Analysis of the bone marrow cytokine and chemokine environment revealed an attenuated type I and type II interferon response in the mice infected with the persistent strain compared to the self-healing strain, while both strains induced a rapid upregulation of myelopoietic cytokines, such as IL-1β and GM-CSF. These results demonstrate that an active infection in the bone marrow is not necessary for the induction of infection-adapted myelopoiesis, and underline the importance of considering alterations to the bone marrow output when analyzing in vivo host-pathogen interactions.

## 1. Introduction

Hematopoietic stem cells (HSC) are the architects of definitive hematopoiesis; that is, the production of blood cells that occurs continuously throughout the life of an organism. While long-term repopulating HSCs remain mostly dormant, more mature multipotent progenitor cells (MPP) proliferate and ultimately give rise to terminally differentiated mature blood cells: red blood cells that allow efficient transport of oxygen; megakaryocytes and their platelet offspring that interact with the blood vessels and soluble factors to regulate coagulation; and white blood cells of the innate and acquired immune system that defend the organism against pathogens [1,2]. Hematopoiesis occurs in the adult bone marrow (BM) where HSCs reside within a specialized microenvironment, or niche. The BM niche is essential for the regulation of many stem cell activities, including self-renewal, mobilization, and lineage differentiation [3,4,5].

Blood cell production is well balanced in the absence of external stressors, with the various subsets of MPP each contributing to the BM output. However, this equilibrium shifts toward myelopoiesis during infection, either in response to pro-inflammatory cytokines or directly via the recognition of pathogen-associated molecular patterns by the progenitor cells [6,7,8,9,10,11]. This BM response to inflammation, also known as emergency myelopoiesis, involves transient proliferation and expansion of MPP and short-term HSC populations. More specifically, myeloid-biased MPP subpopulations, such as MPP2 (biased toward platelet and red blood cell differentiation) and MPP3, tend to increase in size, while lymphoid production is suppressed and the number of Flt3^+^ lymphoid-biased MPPs goes down. In the case of systemic bacterial infections or the administration of lipopolysaccharide, inflammation-adapted myelopoiesis tends toward granulocyte differentiation in response to the secretion of Granulocyte-colony stimulating factor (G-CSF) by BM endothelial cells [6,12]. However, the role of emergency myelopoiesis in parasitic infections remains less clear.

Visceral leishmaniasis caused by the parasites *Leishmania donovani* and *L. infantum* is a chronic infection, associated with hematological changes, such as anemia and pancytopenia, which indicate impaired BM function. BM is also a well-established site of parasite expansion. Recruitment of CD4^+^ T cells to the BM resulted in increased HSC proliferation and functional exhaustion [13] as well as suppression of erythropoiesis in the mouse model of visceral leishmaniasis [14]. We have also shown that *L. donovani* promotes the production of monocytes and monocyte-derived myeloid cells that not only suppress T cell responses but are also more permissive to infection, thus promoting parasite expansion [15,16]. Monocytes have been long since proposed to act as safe targets for *Leishmania* [17], and the ability of the parasite to not only stimulate monocyte recruitment and production but also to promote the differentiation of cells with impaired microbicidal function [15,16] further supports this idea.

Generating safe harbors for replication is likely not restricted to visceral leishmaniasis, but may also occur in cutaneous leishmaniasis [17]. *L. major*, one of the predominant species causing cutaneous leishmaniasis, most often results in a transient skin lesion that heals within 2–8 months [18]. However, some patients have persistent, non-healing lesions with severe inflammation and scarring. The mechanisms underlying immune resistance in persistent *L. major* infection are still incompletely understood. It is possible to address this issue by comparing the response to two different strains of *L. major* in C57BL/6 mice: *L. major* Seidman (*Lm*Sd) strain that causes non-healing and disseminating lesions; and *L. major* Friedlin (*Lm*Fn) strain that causes self-healing lesions after intradermal injection. Both strains evoke a strong CD4^+^ Th1 response in the skin lesion, but the non-healing strain also promotes a strong migration of cells of myeloid origin to the lesion [19,20].

Based on the association of myeloid recruitment with the establishment of persistent lesions, we thus hypothesized that infection-adapted myelopoiesis could play a role also in cutaneous leishmaniasis. We found that infection of C57BL/6 mice with the non-healing strain resulted in the expansion of MPPs in BM and spleen, and promoted the increase in myeloid cell numbers not only in the skin lesion but also in the spleen. Numbers of BM CD19^+^ B lymphocytes were decreased in mice infected with either strain. Interestingly, the BM cytokine responses were stronger in mice infected with the self-healing strain, and this was especially apparent at the later time points, where interferon-responsive chemokines were significantly decreased in mice with persistent lesions. Put together, our results demonstrate that the presence of the pathogen in the BM is not necessary for the induction of infection-adapted myelopoiesis, but a persistent infection with a cutaneous pathogen can also influence BM function.

## 2. Materials and Methods

### 2.1. Experimental Animals and Parasites

**Figure 1:**
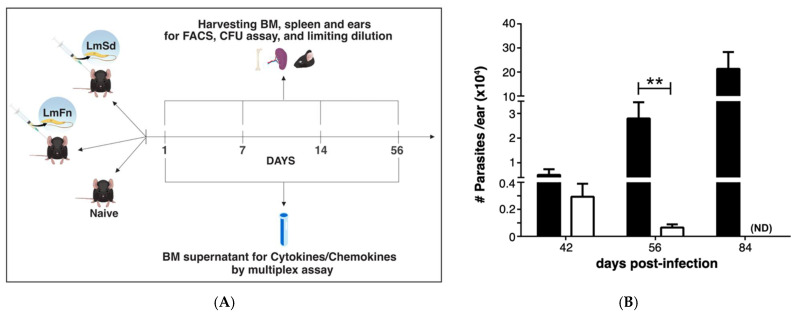
*Lm*Sd and *Lm*Fn infections in C57BL/6 mice: (**A**) Experimental design. C57BL/6 mice were inoculated in the ear dermis with 1 × 10^3^ *Lm*Fn and *Lm*Sd metacyclic promastigotes. Bone marrow, spleen, and infected ears were analyzed on day 1, 7, 14, and 56 post-infection. FACS analysis and culture for parasite burden were performed for all times-points. Cytokine and chemokine analyses were performed on day 1 and day 56 by multiplex assay. (**B**) Parasite burden in the infected ears as determined by limiting dilution analysis (LDA) over the course of infection. No parasites were detected at earlier time points in the ear or at any time point in the bone marrow. Graph represents the mean ± SEM: *n* = 3, *n* = 10 and *n* = 3 mice per group (*Lm*Fn vs. *Lm*Sd) on days 42, 56, and 84, respectively. ** *p* < 0.05 comparing infection with *Lm*Fn and *Lm*Sd.

**Figure 2:**
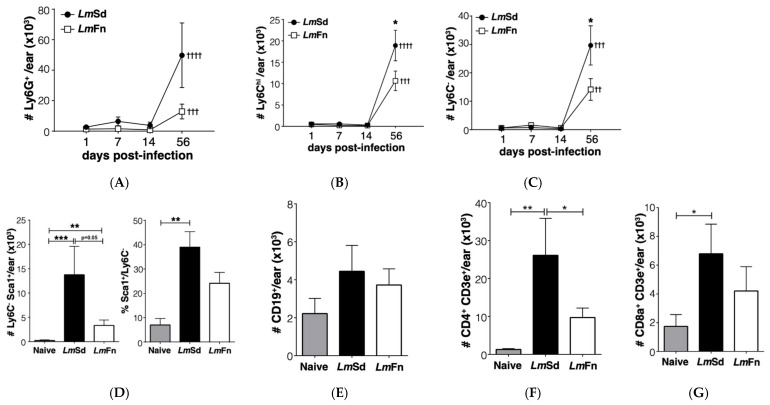
Myeloid cell accumulation at the site of infection in cutaneous leishmaniasis. Populations of myeloid (CD11b^+^) cells were defined by the following markers: (**A**) Ly6G^+^ granulocytes; (**B**) Ly6C^hi^ Ly6G^−^ inflammatory monocytes; and (**C**) Ly6C^−^Ly6G^−^ resident dermal macrophages and monocyte-derived myeloid cells. (**D**) Number and percentage of Ly6C^−^Ly6G^−^MHC-II^+^ cells that express Sca-1 antigen on day 56 post-infection. (**E**) CD19^+^ B lymphocytes, (**F**) CD4^+^ and (**G**) CD8^+^ T lymphocytes detected on day 56 post-infection. Graphs represent compiled results (mean ± SEM) from at least 4 independent experiments for each time point, with *n* = 9, *n* = 7, *n* = 7 and *n* = 15 mice per group (*Lm*Fn vs. *Lm*Sd) at 1-, 7-, 14-, and 56-days post-infection, respectively. *, **, *** *p* < 0.05 comparing infection with *Lm*Fn vs. *Lm*Sd at a given time point and ^††, †††, ††††^ *p* < 0.05 comparing infected groups (*Lm*Fn or *Lm*Sd) to naïve control mice. See also Supplementary Figure S1 for flow cytometry gating strategies and representative results.

**Figure 3:**
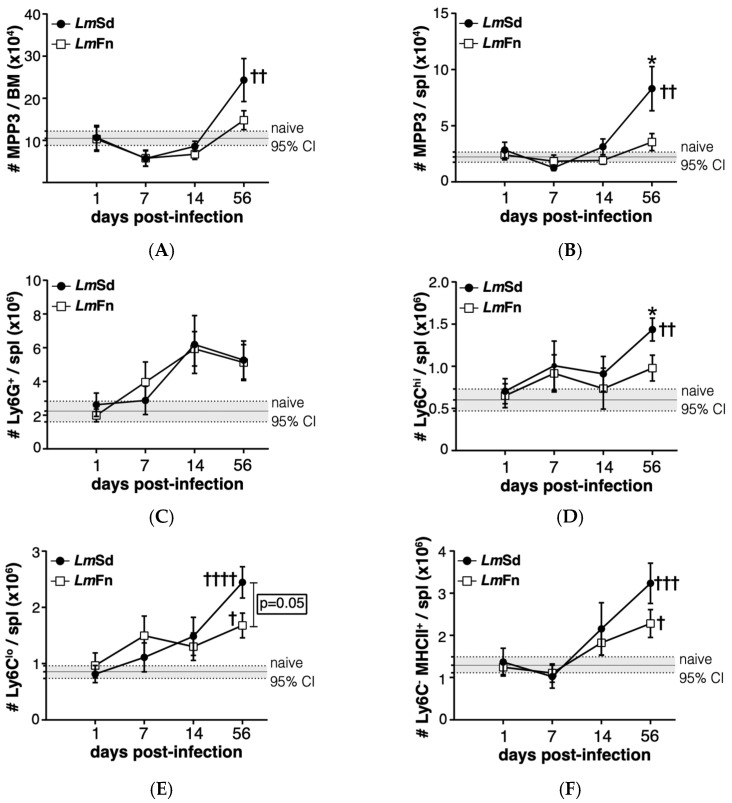
Induction of myeloid-biased multipotent progenitors is associated with an accumulation of myeloid cells during persistent infection with *L. major*. The number of cells within the CD48^+^CD150^−^CD135^−^ MPP3 subset of hematopoietic progenitor cells in (**A**) bone marrow and (**B**) spleen at different times post-infection. The number of (**C**) Ly6G^+^ granulocytes; (**D**) Ly6C^hi^Ly6G^−^ inflammatory monocytes; (**E**) Ly6C^Lo^Ly6G^−^ alternative monocytes; and (**F**) Ly6C^−^Ly6G^−^MHCII^+^ monocyte-derived myeloid cells in the spleen. Graphs represent compiled results (mean ± SEM) from at least 4 independent experiments for each time point, with *n* = 13, *n* = 11, *n* = 11, and *n* = 15 mice per group (*Lm*Fn vs. *Lm*Sd) at 1-, 7-, 14-, and 56-days post-infection, respectively. The grey horizontal line and shaded area represent mean ± 95% confidence interval for naïve control mice (*n* = 16). * *p* < 0.05 comparing infection with *Lm*Fn vs. *Lm*Sd at a given time point and ^†, ††, †††, ††††^ *p* < 0.05 comparing infected groups (*Lm*Fn or *Lm*Sd) to naïve control mice. See also Supplementary Figures S2 and S3 for flow cytometry gating strategies and representative results.

**Figure 4:**
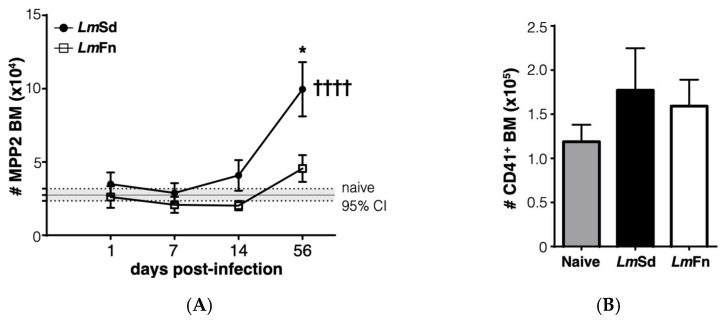

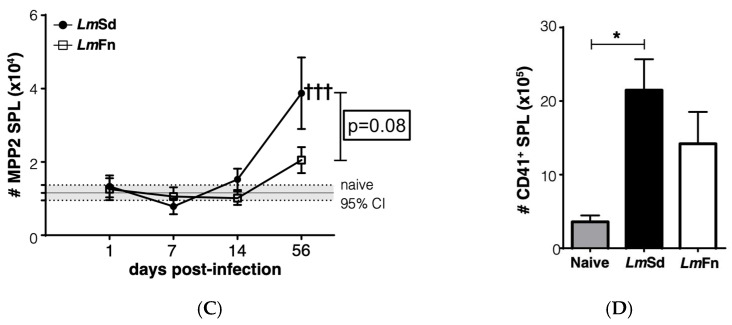
Accumulation of platelet-biased multipotent progenitors in bone marrow and spleen in *Lm*Sd-infected mice: (**A**) The number of cells within the CD48^+^CD150^+^CD135^−^ MPP2 subset of hematopoietic progenitor cells in the bone marrow at different times post-infection. (**B**) Analysis of the number of CD45^−^CD41^+^ platelets in the bone marrow on day 56 post-infection. (**C**) Number of MPP2 cells in the spleen at different times post-infection. (**D**) Analysis of the number of CD45^−^CD41^+^ platelets in the spleen on day 56 post-infection. Graphs represent compiled results (mean ± SEM) from at least 4 independent experiments for each time point, with *n* = 13, *n* = 11, *n* = 11, and *n* = 15 mice per group (*Lm*Fn vs. *Lm*Sd) at 1-, 7-, 14-, and 56-days post-infection, respectively, for MPP2 analysis; and from 2 independent experiments with *n* = 7 mice per group for platelet analysis. The grey horizontal line and shaded area in panels (**A**,**C**) represent mean ± 95% confidence interval for naïve control mice. * *p* < 0.05 comparing infection with *Lm*Fn vs. *Lm*Sd at a given time point and ^†††, ††††^ *p* < 0.05 comparing infected groups (*Lm*Fn or *Lm*Sd) to naïve control mice. See also Supplementary Figures S2 and S4 for flow cytometry gating strategies and representative results.

**Figure 5:**
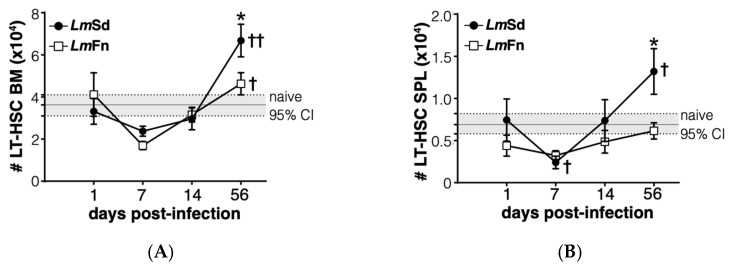
Long-term HSC-like cells display biphasic kinetics in bone marrow and spleen of *Lm*Sd-infected mice. Number of cells expressing LT-HSC markers in (**A**) bone marrow and (**B**) spleen at various time points. Graphs represent compiled results (mean ± SEM) from at least 4 independent experiments for each time point, with *n* = 13, *n* = 11, *n* = 11, and *n* = 15 mice per group (*Lm*Fn vs. *Lm*Sd) at 1-, 7-, 14-, and 56-days post-infection, respectively. The grey horizontal line and shaded area represent mean ± 95% confidence interval for naïve control mice. * *p* < 0.05 comparing infection with *Lm*Fn vs. *Lm*Sd at a given time point and ^†, ††^ *p* < 0.05 comparing infected groups (*Lm*Fn or *Lm*Sd) to naïve control mice. See also Supplementary Figure S2 for flow cytometry gating strategies and representative results.

**Figure 6:**
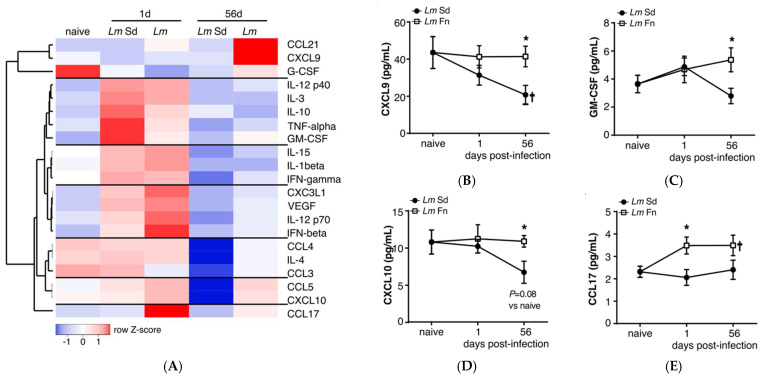
Bone marrow inflammatory cytokine/chemokine profiles indicate early activation and subsequent suppression of inflammatory responses in non-healing *L. major* infection: (**A**) Soluble cytokine/chemokine levels in bone marrow extracellular milieu after *Lm*Fn and *Lm*Sd infection. Heatmap represents relative quantities for each cytokine/chemokine as fold change compared to paired, sex-matched naive mice, and normalized across each row. Each square represents the average fold change for a sample of 4 males and 4 females mice per group. The raw concentration of (**B**) CXCL-9 (MIG); (**C**) Granulocyte-macrophage colony-stimulating factor (GM-CSF); (**D**) CXCL-10 (IP-10); and (**E**) CCL-17 (TARC) in bone marrow supernatant obtained from naïve and infected mice at 1- and 56-days post-infection. Graphs represent the mean ± SEM. * *p* < 0.05 comparing infection with *Lm*Fn vs. *Lm*Sd at a given time point and ^†^ *p* < 0.05 comparing infected groups (*Lm*Fn or *Lm*Sd) to naïve control mice.

All experimental mice were wild-type littermates from our breeding colonies on C57BL/6 background, and maintained under specific pathogen-free conditions in ventilated racks. Both male and female mice were used for the study with sex- and age-matched controls. For infections, mice were transferred to the biosafety level 2 sector at the animal facility of INRS (Laboratoire national de biologie expérimentale, LNBE, Laval, QC, Canada). Promastigotes of *L*. *major* NIH S (MHOM/SN/74/Seidman) and *L*. *major* (MHOM/IL/80/Friedlin) were cultured at 26 °C in *Leishmania* medium (M199 medium supplemented with 10% heat-inactivated FBS, 100 μm hypoxanthine, 10 mm HEPES, 5 μm hemin, 3 μm biopterin, 1 μm biotin, and penicillin–streptomycin) [21]. Infectious-stage metacyclic promastigotes were isolated from stationary cultures (5–6 days) by density gradient centrifugation [22] and mice were then inoculated with 1 × 10^3^ metacyclic promastigotes in the ear dermis by intradermal (i.d.) injection in a volume of 20 μL [23,24,25]. Lesion progression and weight loss were monitored weekly, and the mice were euthanized under CO_2_ asphyxiation at indicated time points (Figure 1A). There was a total of 48 mice infected with *Lm*Sd (25 males and 23 females), 46 mice infected with *Lm*Fn (24 males and 22 females), and 20 naïve controls (10 males and 10 females) analyzed at the time points presented in Figure 2, Figure 3, Figure 4, Figure 5 and Figure 6. Analysis of parasite burden from two additional groups of mice was added for days 42 and 84 to evaluate the establishment of early infection, and to confirm remission of the self-healing parasite, respectively.

### 2.2. Ethics Statement

All procedures were in accordance with the Canadian Council on Animal Care guidelines and approved by the Comité institutionnel de protection des animaux of the INRS (CIPA # 1706-07 approved in August 2017; 1806-01, August 2018; and 1811-02, December 2018).

### 2.3. Processing of Ear Tissues and Evaluation of Parasite Burden

Infected mouse ears were collected and incubated in 70% ethanol for 10 min. The two sheets of infected ear dermis were separated, deposited in DMEM containing 100 U/mL penicillin, 100 μg/mL streptomycin, and 0.2 mg/mL Liberase CI purified enzyme blend (Sigma-Aldrich Canada, Oakville, ON, Canada), and incubated for 1.5 h at 37 °C [23]. Digested tissue was processed in a tissue homogenizer (Medimachine; BD Biosciences, San Diego, CA, USA) and filtered through a 70 μm cell strainer (Falcon Products, Newport, TN, USA). Parasite titrations were performed using tissue homogenates, which were serially diluted in 96-well flat-bottom microtiter plates containing *Leishmania* medium (M199 medium supplemented with 10% heat-inactivated FBS, 100 μm hypoxanthine, 10 mm HEPES, 5 μm hemin, 3 μm biopterin, 1 μm biotin, and penicillin-streptomycin) and cultured at 26 °C. The number of viable parasites in each ear was determined from the highest dilution at which promastigotes could be grown out after 7–10 days of incubation [26]. Data are presented as number of parasites per ear. The same assay was performed on freshly harvested BM from infected mice with no parasite growth observed even after 14 days of incubation.

### 2.4. Flow Cytometry

BM, spleen, and cells recovered from the ear dermis from infected and naïve mice were analyzed by flow cytometry. BM was harvested by flushing tibiae and femora in phosphate-buffered saline (PBS) solution and the cells were then passed through a 25-gauge needle to obtain a single cell suspension. Spleens were manually dissociated in PBS. Ear tissue homogenates, prepared as described above, were filtered in 5 mL round bottom polystyrene FACS tubes with 35 μm nylon mesh-screen filter before flow cytometry staining. PBS was supplemented with 0.1% bovine serum albumin (BSA) and 0.5 mm ethylene-diaminetetraacetic acid (EDTA) for flow cytometry staining, and non-specific staining was blocked using unlabeled anti-CD16/CD32 antibody (Fc block). Antibody combinations were modified from previously published studies, including by our group [16,27,28,29]. Please see Table S1 for a complete list of antibodies. Cells were fixed with 2%PFA in PBS prior to acquisition of samples using a 4-laser LSR Fortessa (BD Biosciences, Mountain View, CA, USA). Data were analyzed using *FACS DiVa* software (version 8.1).

### 2.5. Bone Marrow Cytokine/Chemokine Analysis

Bone marrow cell supernatants were collected from naïve and *L. major-*infected mice on day 1 and eight weeks (56 days) post-infection by harvesting cells from both hind limbs by flushing with 2 mL PBS, followed by centrifugation. The supernatants (diluted bone marrow plasma) were transferred in clean tubes, identified and aliquoted, and then kept frozen until analysis. Cytokine and chemokine concentrations were determined by the Mouse Cytokine 44-Plex Discovery Assay (Eve Technologies, Calgary, AB, Canada) from duplicate analysis of eight mice per group (4 males and 4 females). Please see Supplementary Table S2 for a complete list of cytokines and chemokines in the assay. Heat map was generated for the mean values for each condition, paired, and adjusted by sex, using the Heatmapper online tool [30].

### 2.6. Statistical Analysis

Statistical significance was determined using unpaired ANOVA (for multiple comparisons) or two-tailed student’s *t* test and the *GraphPad Prism* software (version 7).

## 3. Results

### 3.1. Myeloid Cells Recruited to the Skin Lesions at Late Time Points during Persistent Leishmania major Infection Adopt a Regulatory Phenotype

The development of non-healing skin lesions in C57BL/6 mice infected with the *Lm*Sd strain has been associated with enhanced recruitment of myeloid cells to the dermis [19], which led us to hypothesize that persistent cutaneous leishmaniasis would induce an adaptive response in the BM, similar to what we and others have previously reported in experimental visceral leishmaniasis [13,16]. To test our hypothesis, we compared *Lm*Sd to the self-healing *Lm*Fn strain using the model of intradermal infection in the ear (Figure 1A). The number of parasites in the ear remained below the detection limit at the early time points (up to 14-days post-infection (p.i.)), and more importantly, no viable parasites were observed at any time point in the BM, even after 14 days of incubation. Parasites from both strains were able to establish an initial infection as seen by comparable parasite load on day 42 (or 6 weeks p.i.; Figure 1B). While the non-healing infection continued to progress through the later time points, parasite burden for the self-healing *Lm*Fn strain was on decline by day 56 (or 8 weeks p.i.), and there were no detectable parasite remaining by day 84 (or 12 weeks p.i.; Figure 1B). While the lesions caused by *Lm*Fn were visible at 6 weeks p.i., they remained constrained in size and were cleared by late time points as expected. In contrast, lesions from mice infected with *Lm*Sd started to show ulceration and spread by day 84 p.i. These results follow closely what has been previously published by the Sacks group [19] and thus validated our model. We thus decided to focus our study on the early time points (first two weeks) as well as the 8-week time point when the parasite was still detectable but on the decline in the self-healing lesions.

To confirm the accumulation of myeloid cells in non-healing lesions in our model, we analyzed the leukocyte populations in tissue homogenates prepared from the infected ears at different time points. Myeloid cells were initially identified as CD11b^+^ cells and then further classified based on their expression of Ly6C and Ly6G (Figure S1A). In this way, Ly6C^low^ Ly6G^+^ cells represent granulocytes/neutrophils; Ly6C^hi^ Ly6G^−^ cells are mostly inflammatory monocytes; and Ly6C^−^Ly6G^−^ cells represent mostly monocyte-derived myeloid cells (dendritic cells and macrophages). We observed an increase in all CD11b^+^ cell subsets on day 56 p.i. for both strains as compared to uninfected controls (Figure 2A–C). There was considerable variability in the number of neutrophils recovered from *Lm*Sd-infected ears (Figure 2A); however, these results still dovetail with those found in the literature. Moreover, there was a significant increase in the numbers of both monocytes (Figure 2B) and monocyte-derived myeloid cells (Figure 2C) in the skin of *Lm*Sd-infected mice when compared to those infected with *Lm*Fn. We further evaluated the expression of markers associated with altered myeloid cell function, and identified a subset of Ly6C^−^Ly6G^−^ CD11b^+^ cells that expressed Sca-1 (Figure 2D). This subset was present in much higher numbers in mice infected with the non-healing *Lm*Sd strain. These cells also expressed higher levels of the Transferrin receptor CD71 than their Sca-1^−^ counterparts (Figure S1A), and they were all MHCII^+^.

We also analyzed lymphocytes in the infected ears on day 56 p.i. and found no significant alteration in CD19^+^ B lymphocyte numbers for either strain (Figure 2E). Conversely, there was a strong increase in both CD4^+^ (Figure 2F and Figure S1B) and CD8^+^ T lymphocytes (Figure 2G and Figure S1B) as expected, especially for the persistent *Lm*Sd strain. Put together, these results further validate our model, demonstrating a significant accumulation of myeloid cells in the infected ear at the late time point.

### 3.2. Myeloid-Biased Multipotent Progenitor Cells Accumulate in BM and Spleen in Mice Infected with the Non-Healing Strain of L. major

MPPs represent progenitor cells that provide only transient reconstitution when transferred into an immunocompromised host but that are still able to differentiate into most, if not all, blood cell lineages, albeit at variable efficacy. These cells can be identified within the lineage^-^Sca-1^+^c-Kit^+^ (LSK) hematopoietic stem/progenitor cell population according to their expression of surface markers CD150, CD48, and CD135/Flt3 (Figure S2A) [27]. The CD48^+^ CD150^−^ CD135^−^ MPP3 subpopulation is the one that is most strongly associated with monocyte and granulocyte fates both at steady state and under regenerative conditions, including during infection-adapted myelopoiesis [6,10,27]. We were therefore very encouraged to observe an increase in the number of the MPP3 subset in the BM of mice infected with the non-healing *Lm*Sd strain on day 56 p.i. (Figure 3A). There was an initial decrease in MPP3 numbers in the BM of infected mice on day 7 p.i., irrespective of parasite strain; however, the later increase above baseline was restricted to mice infected with the non-healing strain, suggesting that the long-term stress response was specific for the persistent infection. A similar increase was also observed in the spleen of *Lm*Sd-infected mice (Figure 3B).

To better understand whether the putative increase in myeloid differentiation as seen by the increase in myeloid-biased MPP3 numbers would promote the accumulation of myeloid cells at sites other than the infected ears, we analyzed the major myeloid subsets in BM and spleen (Figure S3). There were no significant differences in the number of Ly6C^hi^ monocytes or Ly6G^+^ granulocytes in the BM between naïve and infected mice, independent of the parasite strain (Figure S3A). However, there was a significant increase in myeloid cells in the spleen of *Lm*Sd-infected mice on day 56 p.i., with an emphasis on monocytes and monocyte-derived myeloid cells (Figure 3C–F). Ly6G^+^ granulocytes tended to increase in the spleen of infected mice, irrespective of parasite strain; however, there was considerable mouse-to-mouse variability, and the difference did not meet statistical significance (Figure 3C). In contrast, there were significant differences between the groups of mice infected with *Lm*Sd and *Lm*Fn when it came to Ly6C^hi^ inflammatory monocytes (Figure 3D) or Ly6C^low^ alternative monocytes (Figure 3E) with an increase in the number of Ly6C-expressing cells in the spleens of *Lm*Sd-infected mice as compared to those infected with the self-healing strain or to uninfected controls. There were also more Ly6C^−^ MHCII^+^ monocyte-derived myeloid cells on day 56 p.i. in *Lm*Sd-infected mice as compared to uninfected controls (Figure 3F). The increase in myeloid cells in the spleen was at least in part driven by an increase in total spleen cellularity in mice infected with the persistent *Lm*Sd strain (2.3 × 10^8^ ± 0.3 × 10^8^ for *Lm*Sd as compared to 1.3 × 10^8^ ± 0.2 × 10^8^ for naïve and 1.5 × 10^8^ ± 0.2 × 10^8^ for *Lm*Fn; *p* < 0.05). These results suggest that the increase in MPP3 observed in the BM and spleen of *Lm*Sd-infected mice on day 56 p.i. results in enhanced myeloid differentiation that can be translated into an accumulation of myeloid progeny not only at the site of infection but also in peripheral organs, such as spleen.

### 3.3. Persistent Cutaneous Leishmaniasis Promotes the Accumulation of Stem-like Megakaryocyte Progenitors

Another stem/progenitor cell subset that normally expands during the BM response to inflammation is the CD48^+^CD150^+^ MPP2 subpopulation that contributes to the formation of red blood cells and platelets [27,31]. It has also been shown to contribute to myelopoiesis in response to inflammation. The BM response to the non-healing *L. major* infection was effectively not restricted to the expansion of MPP3 cells, as we could also observe a significant increase in the numbers of cells within the MPP2 subset in the BM on day 56 p.i. (Figure 4A and Figure S2). Similar to myeloid cells, there was no significant increase in platelet numbers in the BM (Figure 4B and Figure S4A), and the numbers of immature erythrocytes tended to decrease (Figure S4A). However, the increase in MPP2 cells in the spleen of *Lm*Sd-infected mice (Figure 4C) was associated with an increased number of platelets (Figure 4D), suggesting that platelet production could also be enhanced in response to infection with *L. major*. There was also an increase in the proportion of immature CD71^+^ erythrocytes in the spleen of infected mice (Figure S4B), and this increase tended to be more significant in mice infected with *Lm*Sd.

The MPP4 subset of CD150^−^ CD48^+^ CD135^+^ LSKs are also known as lymphoid-primed MPPs due to their enhanced potential for efficient differentiation into lymphocytes [32]. However, their frequency and function often decline in conditions of chronic inflammation [16,27]. The MPP4 subset tended to decrease at the early time points (day 7 and day 14), especially in mice infected with the self-healing strain (Figures S2 and S5A). MPP4 numbers mostly recovered by day 56 p.i.; although, there were quite large variations between individual animals. Despite the recovery of MPP4 numbers, there was a decrease in the numbers of CD19^+^ lymphocytes in the BM of infected mice as compared to naïve controls, independently of the type of lesion (Figure S5B,D). Moreover, the proportion of IgM^−^ B lymphocyte precursors as compared to IgD^+^ recirculating mature cells tended to decrease in mice infected with *Lm*Sd, further suggesting a decrease in B lymphopoiesis. There was no difference in CD4^+^ lymphocyte numbers in the BM (Figure S5C,D); however, the number of CD8^+^ lymphocytes was specifically decreased in the BM of *Lm*Sd-infected mice (Figure S5C,D).

Lastly, we wanted to evaluate if the most immature stem/progenitor cell subsets were also affected by the persistent infection. The CD48^−^CD150^+^ LSK subpopulation is enriched in long-term HSCs at steady state [33], but can be contaminated by activated downstream progenitor cells during inflammation [27,31,34]. Similar to the different MPP subpopulations (Figure 3A, Figure 4A and Figure S2), the number of cells corresponding to the HSC phenotype was increased in the BM of mice infected with the non-healing *Lm*Sd strain on day 56 p.i. (Figure 5A). This was also true in the spleen (Figure 5B). Unexpectedly, we also observed a decrease in HSC numbers on day 7 p.i. in both organs and for mice infected with both strains of *L. major* (Figure 5A,B), similar to our results for the MPP3 subset in the BM (Figure 3A). Collectively these results suggest an inflammatory response in the BM that occurs early on in *L. major*-infected mice irrespective of parasite strain, similar to what normally occurs in acute infections [6,12,35,36]. However, while the BM returns more or less to normal in mice infected with the self-healing strain by day 56 p.i., the non-healing strain provokes a second phase of BM response that corresponds to the accumulation of myeloid cells, especially monocytes and monocyte-derived cells, at the site of infection, but also in peripheral organs, such as spleen.

### 3.4. BM Soluble Cytokine/Chemokine Profiles Suggest the Absence of Specific Pro-Inflammatory Responses in Persistent Infection

Activation of infection-adapted myelopoiesis can be the result of direct sensing of the pathogen in the BM or a more indirect response to changes in the BM cytokine environment, depending on the situation [3,5,6,12,13,14,37,38,39]. To obtain a global view of the cytokine response in the BM, we analyzed soluble factors available in the BM from the supernatant (or diluted BM plasma) recovered from naïve mice as well as mice inoculated with *L. major* immediately in the early phase (day 1 p.i.) or late in the chronic phase (day 56 p.i.). We evaluated a panel of 44 different cytokines and chemokines (see Table S2 for the complete list), but only those that were reliably detected in at least 75% of the samples and that varied by at least 50% at any given time point are presented in Figure 6A. We further divided these factors in seven groups based on expression patterns. The first group includes factors downregulated on day 1 and whose levels remain low in mice infected with the non-healing *Lm*Sd strain but come back to baseline or increase in those infected with the self-healing *Lm*Fn strain, such as the interferon-responsive chemokine CXCL9 (Figure 6B). Groups 2–4 consist of factors upregulated on day 1 and whose levels decrease by day 56 in both groups with the exception of GM-CSF, which remains upregulated in *Lm*Fn-infected mice (Figure 6C). Some are upregulated slightly more strongly in *Lm*Sd-infected mice (such as TNF-α or IL-10), others in *Lm*Fn-infected mice (such as IL-12 p70 or IFN-β), while others follow very similar patterns for both strains (e.g., IL-1β and IFN-γ). Groups 5 and 6 comprise factors that remain relatively stable on day 1 but are strongly downregulated in the BM of *Lm*Sd-infected mice on day 56 p.i., such as CCL5/RANTES and the interferon-inducible chemokine CXCL10 (Figure 6D). Finally, there is a single chemokine, CCL17, which is upregulated on day 1 only in *Lm*Fn-infected mice, and whose levels remain elevated in this group even on day 56 (Figure 6E). These results tend to support the presence of an early inflammatory response in the BM of mice infected with either strain of parasite, but unfortunately give us relatively little insight into what would promote sustained myelopoiesis on day 56 in mice infected with the non-healing strain. Instead, they point toward a decrease in interferon (CXCL10, CXCL9, IFN-β, and IL-12 p70) and GM-CSF- (GM-CSF, CCL17)-associated responses in the BM of mice infected with the non-healing strain both at early and late time points.

## 4. Discussion

BM hematopoietic progenitor cell activation and the subsequent induction of adapted hematopoiesis to infection and inflammation can have positive or negative consequences on the host immune response and hematopoietic recovery, depending on the type and duration of stimulus [3,6,10,16]. We have previously shown that infection-adapted myelopoiesis promoted parasite expansion during experimental visceral leishmaniasis, namely by enhancing the generation of monocytes that were highly susceptible to infection by the parasite [16] and contributed to the suppression T lymphocyte responses [15]. Here, we wanted to evaluate the hypothesis that the dissemination of parasite to the BM was not strictly required for a hematopoietic response and that a persistent, non-healing infection with the cutaneous species *L. major* would also promote myeloid differentiation. We found that the intradermal infection of C57BL/6 mice in the ear with a non-healing strain of *L. major* resulted in the expansion of myeloid-biased progenitor cells in BM and spleen, in contrast to an infection with a self-healing strain, and promoted the increase in myeloid cell numbers not only in the skin lesion but also in the spleen. Our results further suggest a suppression of inflammatory responses at later time points in mice infected with the non-healing strain and underline the importance of considering alterations to the BM output when analyzing in vivo host-pathogen interactions.

Enhanced myelopoiesis has been suggested to contribute to *Leishmania* parasite proliferation by generating “safe targets”, or permissive cells in which the parasite can hide from the host immune response, for quite some time [17]. While visceral leishmaniasis establishes a chronic infection in the BM and is associated with signs of BM failure if untreated [16,40], the impact of an intradermal cutaneous infection on BM function has not been thoroughly investigated. The initial report by Mirkovich et al. found mild increases in colony-forming activity in the spleen but not in the BM of C57BL/6 mice infected intracutaneously with a large number (5 × 10^6^) of promastigotes [17]. Our results, using a much lower number of parasites, but enriching for the infectious form prior to inoculation, point toward a similar direction, and even though we observe an increase in myeloid-biased progenitor cells in both organs, there was no accumulation of myeloid cells in the BM. These results are very similar to what we have also reported in the mouse model of visceral leishmaniasis [16].

We did not detect any viable parasite in the BM in a limiting dilution assay, using the equivalent of 5 × 10^6^ BM cells, or approximately 10% of cells harvested from two hind legs (femora and tibiae) as starting dilution. This is in contrast to the findings of a previous study based on the intradermal inoculation of a 10-fold higher dose (10^4^) of metacyclic *L. major* promastigotes and on the use of quantitative PCR, where parasite DNA was sporadically detected in the BM of infected B10D2 and BALB/c mice [25]. Even higher doses (5 × 10^4^ metacyclic or 2 × 10^6^ stationary phase promastigotes) have also been shown to yield low but detectable numbers of viable parasites in the spleen of C57BL/6 mice [41,42]. However, such high doses are likely not very representative of a natural infection and may result in a different disease course that is cleared more rapidly [43]. Although we cannot completely exclude the possibility that a small number of parasites reach the BM based on a limiting dilution assay alone; and while more sensitive methods, such as quantitative PCR on a high-copy number template, could yield a different result, our data confirm that there is no active infection in the BM. While more sensitive, PCR-based methods are also more likely to overestimate the amount of viable pathogen and may detect left-over genetic material from an aborted infection or a recent contact with the pathogen that has been cleared [44,45]. More specifically, in the context of our study, cells such as neutrophils [46,47] or migratory dendritic cells [42] could also bring phagocytosed parasite material from skin or draining lymph nodes to the BM and spleen. Neutrophil clearance in the BM also stimulates further granulopoiesis [47], which would contribute to the expansion of myeloid-biased progenitor cells in persistent infection; however, this remains a hypothesis in the context of cutaneous leishmaniasis.

While the contribution of neutrophils and dermal macrophages to the non-healing lesions in *Lm*Sd-infected mouse ear has been previously described [19,20], the importance of infiltrating monocytes has remained more obscure. Monocytes become rapidly infected by *L. amazonensis*, another *Leishmania* species causing cutaneous leishmaniasis [48], and their presence is required for early parasite expansion in this model. While the non-healing strain of *L. major* preferentially infects dermal macrophages that are independent of circulating monocytes at the early time points, monocytes are also infected to a greater extent than with the self-healing strain [20]. Moreover, the contribution of monocytes to the maintenance of dermal macrophages at later time points (after more than 1 month) cannot be completely excluded, as experiments using BM chimeras showed approximately 25% contribution of BM-derived donor cells to the dermal macrophage compartment at 16 weeks [20]. Finally, a recent publication using another strain of *L. major* demonstrated that parasite replication in self-contained infection was limited by decreased monocyte recruitment due to NO [49]. The association of enhanced monocyte accumulation in the periphery and increased numbers of myeloid progenitor cells with persistent infection in our study further suggests that monocytes contribute to parasite replication in cutaneous leishmaniasis. We also identify a subset of Sca-1^+^ CD71^+^ MHC-II^+^ myeloid cells in the ear dermis that is quite specific for mice infected with the non-healing *Lm*Sd strain, and we speculate that it represents a monocyte-derived subset that has upregulated Sca-1 expression in situ in response to infection, as we have previously observed that *Leishmania* infection alone was sufficient to promote Sca-1 expression in vitro [16]. Upregulation of Sca-1 and MHC-II expression by monocytes has also been associated with the acquisition of regulatory function in *Toxoplasma gondii* infection [38], while Sca-1^+^ myeloid cells contribute to tissue damage and mortality in experimental infection with *Staphylococcus aureus* [50]. CD71 expression has been linked to activated monocytes as well as to tissue macrophages, and elevated CD71 levels have been reported, for example, in Post Kala-Azar dermal leishmaniasis patients, both in skin lesions and in circulating monocytes [51]. Further analyses will be required to evaluate the functional importance of this subset.

Control of cutaneous leishmaniasis is highly dependent on early, efficient Th1 responses in the skin and draining lymph nodes [42,52,53]. An ineffective Th1 response, such as seen in immunosuppressed individuals [54], genetically modified mouse strains [42], or in the susceptible Balb/c mouse strain [52,53] results in systemic spread, especially in the context of a high parasite load. Lymphocyte population in the dermis remains mostly Th1-biased even in the persistent *L. major* infection [20]; however, cytokine production in this case was evaluated by intracellular staining of PMA/ionomycin restimulated lymphocytes and, thus, more accurately represents potential rather than actual production in vivo. Neutrophil- and monocyte-derived myeloid suppressor cells can inhibit pathogen-specific lymphocyte responses and contribute to tissue damage [15,50,55], and could thus contribute to promoting parasite persistence and the development of skin lesions in persistent infection. Moreover, the production of regulatory myeloid cells or cells more permissive to infection, as reported in visceral leishmaniasis [16] or in toxoplasmosis [38], for example, would not only interfere with Th1 responses but also provide more target cells for the parasite, as previously discussed. This would not diminish the importance of resident dermal macrophages as primary targets for the persistent *Lm*Sd strain [20], but provide additional “safe houses”. Altered myelopoiesis in the BM is also likely to suppress dendritic cell differentiation [56], and could thus attenuate local T lymphocyte responses in the dermis and draining lymph nodes [57].

Another question that remains yet unanswered is the mechanism by which persistent *L. major* infection would promote myelopoiesis. The concentration of most myeloid growth factors in the BM environment was quite low in our assay, especially in the late phase of the infection, but we cannot exclude the possibility that these factors are being consumed by the developing myeloid cells as soon as they are being produced, leaving only low amounts available in the soluble, extracellular milieu. We cannot thus presume that the BM environment would be lacking in cytokines necessary for myeloid differentiation. In vitro colony forming assays showed mild increases in monocytic colonies and total myeloid colonies from the BM of infected mice as compared to naïve controls on day 56 p.i. (Figure S6), suggesting an increase in the number of functional myeloid progenitors in the form of cytokine-responsive cells with robust proliferative potential; however, there was no difference between mice infected with the different strains. Continuous release of newly differentiated myeloid cells from the BM to circulation might also not allow their accumulation in the BM despite their enhanced synthesis. In contrast to the BM, monocytes and monocyte-derived cells accumulated in the spleen of mice infected with the non-healing *Lm*Sd strain. This accumulation was also accompanied by an increase in myeloid-biased progenitor cells, suggesting that some of these cells could indeed be the result of myeloid differentiation in the spleen. It has been shown in the hamster model of visceral leishmaniasis that at least some of the myeloid cells accumulating in the spleen could be the result of intrasplenic proliferation [58]; however, this does not exclude the potential contribution of enhanced myelopoiesis as immature cells are more likely to proliferate than mature myeloid cells. There was also an accumulation of platelets and an increase in overall spleen cellularity in mice infected with the non-healing *Lm*Sd strain, further suggesting that spleen could be a site of active hematopoiesis in persistent cutaneous leishmaniasis. Extra-medullary hematopoiesis is common when there is chronic inflammation in the BM, and the specific export of progenitor cells with high potential for megakaryopoiesis, or the production of platelets, has been reported in response to acute inflammatory signaling to promote platelet recovery [31].

HSC activation and enhanced monopoiesis in visceral leishmaniasis have been attributed to IFN-γ-producing CD4^+^ T lymphocytes being recruited to the BM [13,59]. We observed no accumulation of CD4^+^ T lymphocytes in the BM of either *Lm*Sd- or *Lm*Fn-infected mice, but this does not on its own prove that there could not be a specific increase in IFN-γ-producing cells, even if free IFN-γ levels were very low in BM plasma. However, there was a significant decrease in IFN-γ-responsive factors, such as CXCL9/MIG and CXCL10/IP-10, in the BM of *Lm*Sd-infected mice, suggesting that response to IFN-γ was attenuated in persistent infection. The role of IFN-γ in myelopoiesis remains context dependent, which makes it difficult to interpret the functional importance of our finding. On one hand, IFN-γ is important for macrophage priming and microbicidal effector responses, including the upregulation of iNOS, a key enzyme for the control of leishmania proliferation [41,52,53,60,61]. *Leishmania* parasites are also well known for their ability to suppress host cell responsiveness to IFN-γ to promote their survival inside infected macrophages [62,63]. Reduced IFN-γ responses in the BM, specifically in mice infected with the persistent strain, would thus indicate reduced ability to control the parasite, particularly if this reduced responsiveness is transported to the skin dermis. On the other hand, early exposure to IFN-γ during myeloid differentiation may also have a negative impact on monocyte function, as shown by the induction of regulatory monocytes in the mouse model of toxoplasmosis [38]. Future studies examining the functional role of myeloid progenitors in BM and spleen of *Lm*Sd-infected mice should allow us to address these questions.

In conclusion, our results using the intradermal inoculation of *L. major* as model of cutaneous leishmaniasis show the accumulation of myeloid-biased progenitor cells and mature myeloid cells in mice infected with the *Lm*Sd strain causing non-healing lesions as compared to mice infected with a self-healing strain or to naïve controls. These results demonstrate that the presence of active, widespread infection in the BM is not required for the induction of an infection-adapted hematopoietic response and emphasize the importance of considering hematopoietic alterations when analyzing in vivo host–pathogen interactions.

## Data Availability

Data presented in this study are available in the article and Supplementary Materials.

## Supplementary Materials

The following supporting information can be downloaded at: https://www.mdpi.com/article/10.3390/microorganisms10030535/s1, Figure S1: Flow cytometry gating strategy and representative results in the infected ear dermis, Figure S2: Flow cytometry gating strategy and representative results for BM stem/progenitor cell subsets, Figure S3: Flow cytometry gating strategy and representative results for myeloid cells in BM and spleen, Figure S4: Flow cytometry gating strategy and representative results for red blood cells and platelets, Figure S5: Lymphocytes in the bone marrow; Figure S6: Myeloid colony-forming units in the bone marrow, Table S1: List of antibodies used for flow cytometry; Table S2: List of cytokines and chemokines in the multiplex assay.
