# Supplementary material for: Persistent Cutaneous Leishmania major Infection Promotes Infection-Adapted Myelopoiesis
Source: Microorganisms. 2022 Feb 28;10(3):535. doi: 10.3390/microorganisms10030535 (PMC8954948; doi:10.3390/microorganisms10030535)
Supplement: Supplementary file 1 [file microorganisms-10-00535-s001.zip › Supplementary figure legends.pdf]

Supplementary Figure Legends

Supplementary Figure S1: Flow cytometry gating strategy and representative results in the infected ear dermis. (A) Representative flow cytometry data for granulocytes ($GR1^{hi}$), inflammatory monocytes ($Ly6C^{hi}CD11b^{+}$) and monocyte-derived myeloid cells ($Ly6C^{-}$ $CD11b^{+}$) and Sca-1 expression. **(B)** Representative flow cytometry data fo $CD3\varepsilon^{+}$ $CD4^{+}$ and $CD3\varepsilon^{+}CD8^{+}$ T cell subsets. All percentages are calculated on the parent population (indicated at the top of the column).

Supplementary Figure S2: Flow cytometry gating strategy and representative results for BM stem/progenitor cell subsets. (A) Gating strategy and representative flow cytometry data for a naïve mouse, demonstrating the identification of Lin^{-} $Sca1^{+}$ $cKit^{+}$ (LSK) stem/progenitor cells and their different subsets according to CD150, CD48, and CD135 expression (MPP3, MPP2, LMPP and LT-HSC) in the bone marrow. The same strategy was also used for the spleen. **(B)** Representative flow cytometry data for infected mice on day 1, day 7, and day 56 post-infection. All percentages are calculated on live singlets.

Supplementary Figure S3: Flow cytometry gating strategy and representative results for myeloid cells in BM and spleen. (**A)** Gating strategy and representative flow cytometry plots of naïve and infected mice (day 56), demonstrating the identification of granulocytes ($GR1^{hi}$), and mature monocytes ($Ly6C^{hi}CD11b^{+}$) in the bone marrow. **(B)** Representative flow cytometry plots and gating strategy of naïve and infected mice (day 56), demonstrating the identification of granulocytes ($GR1^{hi}$), inflammatory monocytes ($Ly6C^{hi}CD11b^{+}$), alternative monocytes ($Ly6Cl^{LO/-}$ $CD11b^{+}$), and monocyte-derived myeloid cells ($Ly6C^{-}$ $CD11b^{+}$ MHC-II^{+}) in the spleen. All percentages are calculated on live singlets.

Supplementary Figure S4: Flow cytometry gating strategy and representative results for red blood cells and platelets. Gating strategy and representative flow cytometry plots of naïve and infected mice (day 56) and for platelets ($CD41^{+}$) and the different stages of red blood cell differentiation (based on CD71 and Ter119 expression) in **(A)** the bone marrow and **(B)** spleen. Percentages for platelets refer to total CD45- cells, while those for erythroid cells are calculated as percentage of total erythroid cells (all four stages).

Supplementary Figure S5: Lymphocytes in the bone marrow. (A) The number of cells within the $CD48^{+}CD150^{-}CD135^{+}$ MPP4 subset of hematopoietic progenitor cells in bone marrow at different times post-infection. **(B)** Number of $CD19^{+}$ B lymphocytes, **(C)** $CD4^{+}$ $CD3\varepsilon^{+}$ T lymphocytes, and $CD8^{+}$ $CD3\varepsilon^{+}$ T lymphocytes in BM on day 56 post-infection. Graphs represent are the mean ± SEM of n=13, n=11, n=11 and n=15 mice per group (*Lm*Fn vs *Lm*Sd) at 1,7, 14 and 56 days post-infection, respectively. * $P<0.05$ comparing infection with *Lm*Fn vs. *Lm*Sd at a given time point and † $P<0.05$ comparing infected groups (*Lm*Fn or *Lm*Sd) to naive control mice. The results are compiled from at least 4 independent experiments for each time point. **(D)** Gating strategy and representative flow cytometry data for naïve and infected mice (day 56). The percentages are calculated on live singlets ($CD19^{+}$ cells, $CD3\varepsilon^{+}$ cells) or on the parent population (B and T cell subsets).

Supplementary Figure S6: Myeloid colony-forming units in the bone marrow. Total number of colonies (light grey, black, white) and monocytic colonies (dark grey) per $1x10^{4}$ bone marrow cells isolated from naïve mice or on day 56 post-infection. Bars represent mean + SEM of four mice per group.
