# Supplementary material for: Persistent Cutaneous Leishmania major Infection Promotes Infection-Adapted Myelopoiesis
Source: Microorganisms. 2022 Feb 28;10(3):535. doi: 10.3390/microorganisms10030535 (PMC8954948; doi:10.3390/microorganisms10030535)
Supplement: Supplementary file 1 [file microorganisms-10-00535-s001.zip › Supplementary Table S1.pdf]

ANTIBODY	FLUOROCHROME	CLONE	COMPANY
CD16/32 (Fc block)	Purified	2.4G2	BD Biosciences
HSC staining			
CD117 (cKit)	PE	2B8	BD Biosciences
CD135 (Flt3)	PerCP-eFluor 710	A2F10	eBioscience
CD150	Alexa Fluor 647	TC15-12F12.2	BioLegend
Sca1 (Ly6A/Ly6E)	PE-Cy7	D7	BD Biosciences
CD11b	Biotin	M1/70	BD Biosciences
CD45R/B220	Biotin	RA3-6B2	BD Biosciences
GR1 (Ly6C/Ly6G)	Biotin	RB6-8C5	BD Biosciences
Ter119	Biotin	TER-119	BD Biosciences
CD48	BV421	HM48-1	BD Biosciences
Streptavidin	V500		BD Biosciences
Overview staining			
IgM	PerCP-eFluor 710	II/41	eBioscience
CD19	PE	1D3	BD Biosciences
CD3e	PE-Cy7	145-2C11	eBioscience
CD11b	Alexa Fluor 647	M1/70	BD Biosciences
IgD	APC	11-26c.2a	BD Biosciences
GR1 (Ly6C/Ly6G)	APC-Cy7	RB6-8C5	BD Biosciences
CD4	APCe780	GK1.5	BD Biosciences
CD8a	V450	53-6.7	BD Biosciences
CD45R/B220	A488	RA3-6B2	BD Biosciences
Myeloid and erythroid staining			
CD11b	A647	M1/70	BD Biosciences

Ly6G	PECy7	1A8	BD Biosciences
Ly6C	PerCP-Cy5.5	HK1.4	eBioscience
MHC Class II (I-Ab)	PE	AF6-120.1	BD Biosciences
Sca1 (Ly6A/Ly6E)	BV711	D7	BD Biosciences
Ter119	Biotin/FITC	TER-119	BD Biosciences
Streptavidin	FITC		BD Biosciences
CD71	BV421	C2 (C2F2)	eBioscience
CD41	PE	MWReg30	BD Biosciences
CD45	PE-CF594	30-F11	BD Biosciences

Supplementary Table S1. List of antibodies used for flow cytometry.
