# Supplementary material for: Persistent Cutaneous Leishmania major Infection Promotes Infection-Adapted Myelopoiesis
Source: Microorganisms. 2022 Feb 28;10(3):535. doi: 10.3390/microorganisms10030535 (PMC8954948; doi:10.3390/microorganisms10030535)
Supplement: Supplementary file 1 [file microorganisms-10-00535-s001.zip › Supplementary Table S2.pdf]

Eotaxin	G-CSF	GM-CSF	IFN-γ
IL-1α[1]	IL-1β	IL-2	IL-3
IL-4[1]	IL-5[1]	IL-6	IL-7
IL-9	IL-10	IL-12 p40	IL-12 p70
IL-13[1]	IL-15	IL-17[1]	IP-10/CXCL10
KC/	LIF[1]	MCP-1/CCL2	M-CSF
MIG/CXCL9	MIP-1α/CCL3	MIP-1β/CCL4	MIP-2/CXCL2
RANTES/CCL5	TNF-α	VEGF	6Ckine/CCL21
EPO[1]	Fractalkine/CX3CL1	IFN-β1	IL-11[1]
IL-16	IL-20[1]	MCP-5/CCL12	MDC/CCL22
MIP-3α/CCL20[1]	MIP-3β/CCL19[1]	TARC/CCL17	TIMP-1

Supplementary Table S2. List of cytokines and chemokines in the multiplex assay.

[1] Not detected in most samples
