# Supplementary figures and images for: Persistent Cutaneous Leishmania major Infection Promotes Infection-Adapted Myelopoiesis
Source: Microorganisms. 2022 Feb 28;10(3):535. doi: 10.3390/microorganisms10030535 (PMC8954948; doi:10.3390/microorganisms10030535)

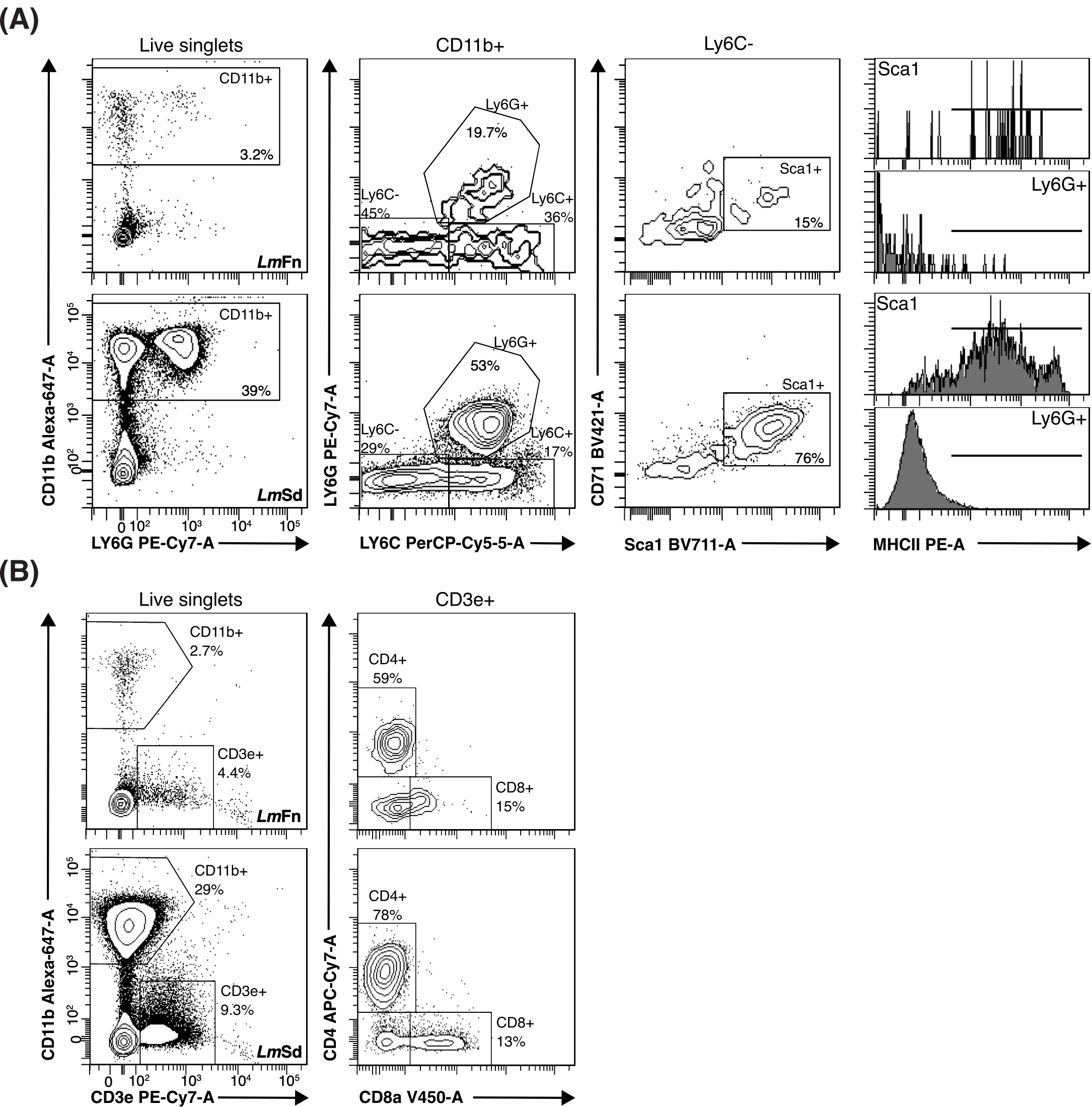

Supplement: Supplementary file 1 [file microorganisms-10-00535-s001.zip › Figure S1.tiff]

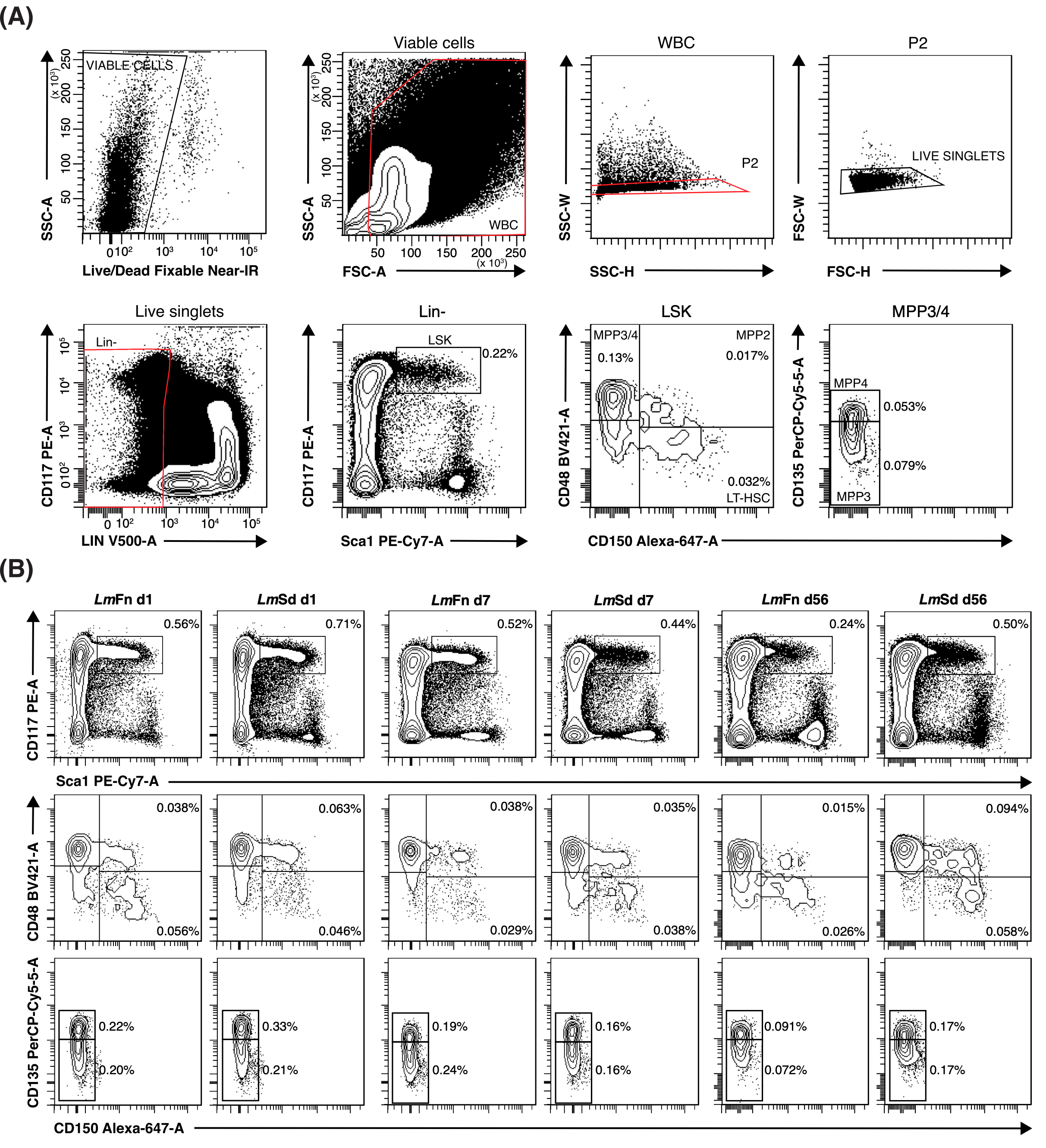

Supplement: Supplementary file 1 [file microorganisms-10-00535-s001.zip › Figure S2.tiff]

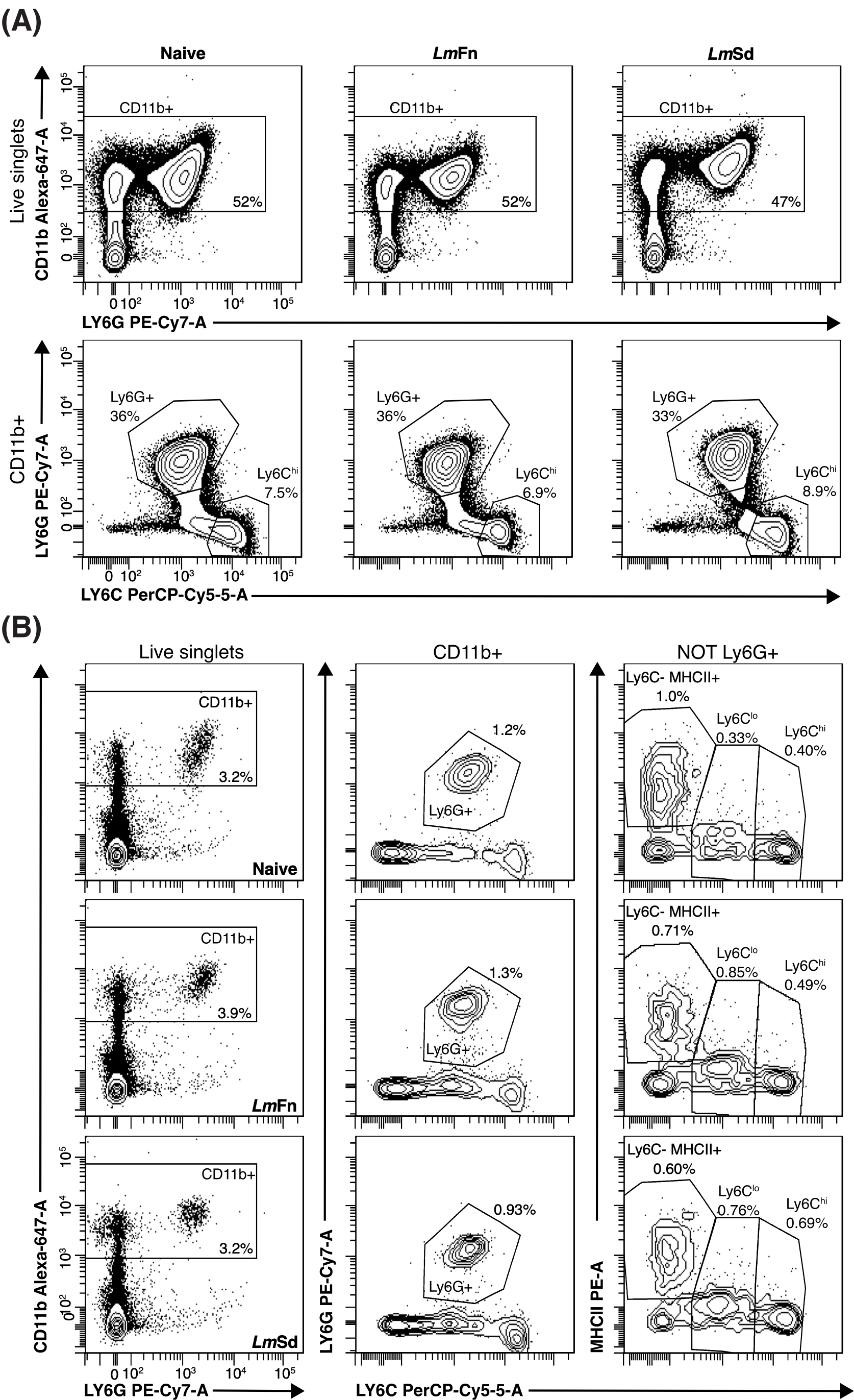

Supplement: Supplementary file 1 [file microorganisms-10-00535-s001.zip › Figure S3.tiff]

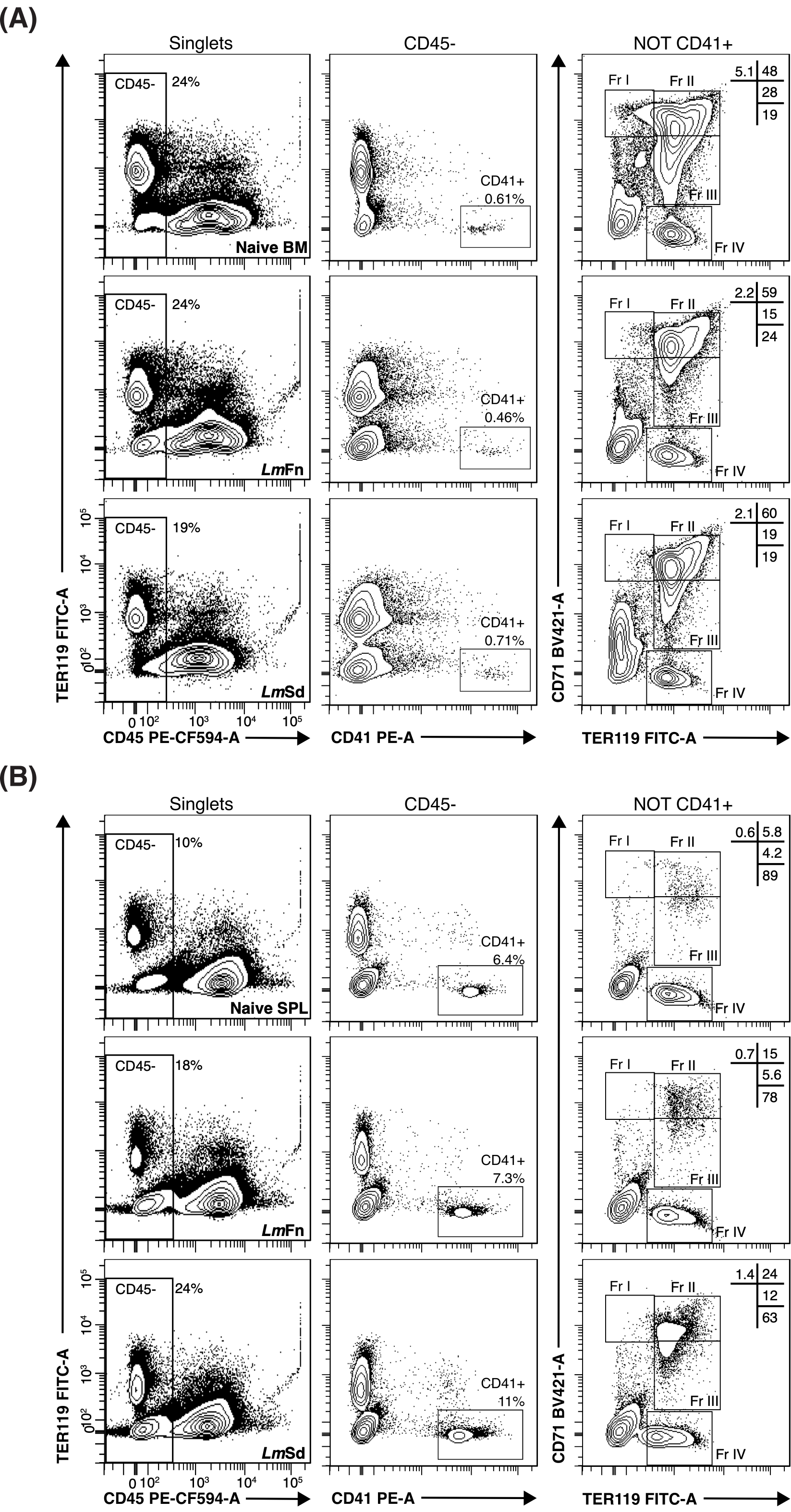

Supplement: Supplementary file 1 [file microorganisms-10-00535-s001.zip › Figure S4.tiff]

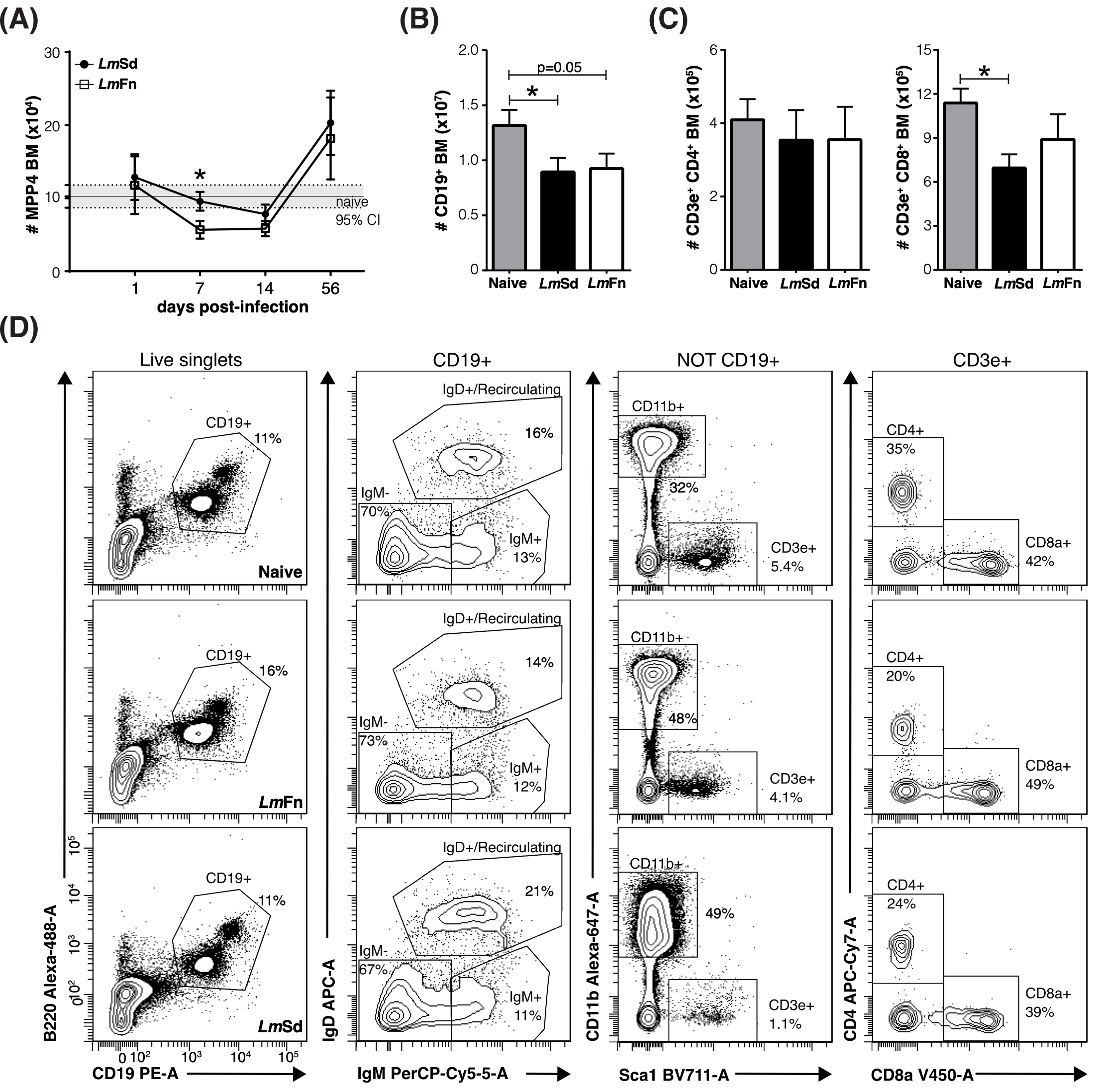

Supplement: Supplementary file 1 [file microorganisms-10-00535-s001.zip › Figure S5.tiff]

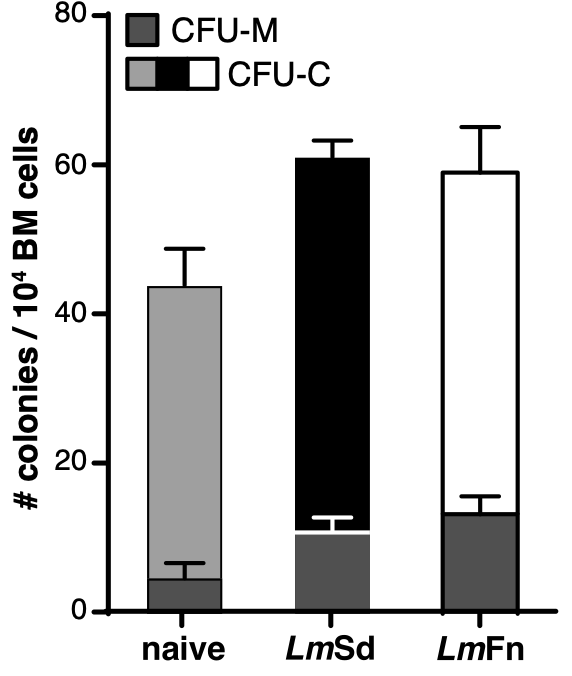

Supplement: Supplementary file 1 [file microorganisms-10-00535-s001.zip › Figure S6.tiff]
